# Atypical presentation of Merkel cell carcinoma with pulmonary embolism

**DOI:** 10.1093/omcr/omaf076

**Published:** 2025-08-20

**Authors:** Romina Garakani, Michael Moradi, Fatima Batool, Muhammad Aameish, Manzoor A Rather

**Affiliations:** Drexel University College of Medicine, 60 N 36th St, Philadelphia, PA 19104, USA; Drexel University College of Medicine, 60 N 36th St, Philadelphia, PA 19104, USA; Department of Internal Medicine, Mercy Fitzgerald Hospital, 1500 Lansdowne Ave, Darby, PA 19023, USA; Department of Internal Medicine, Mercy Fitzgerald Hospital, 1500 Lansdowne Ave, Darby, PA 19023, USA; Department of Internal Medicine, Mercy Fitzgerald Hospital, 1500 Lansdowne Ave, Darby, PA 19023, USA

**Keywords:** Merkel cell carcinoma, metastatic Merkel cell carcinoma, pulmonary embolism

## Abstract

Merkel cell carcinoma (MCC) is a rare aggressive neuroendocrine malignancy that typically presents in the skin and rapidly progresses to other body parts. MCC is typically found in sun-exposed areas, mainly the head and neck region as well as the upper limbs and shoulders. It typically affects fair-skinned elderly males. In this report, we present an unusual MCC case of a 48-year-old Caucasian male with an initial presentation of a massive pulmonary embolism. Further work-up of our patient indicated no typical MCC skin presentation, but rather metastases of the disease. The goal of this report is to highlight the importance of considering MCC as a differential even when patients do not present with common MCC risk factors or skin presentations.

## Introduction

Merkel cell carcinoma (MCC) is a rare neuroendocrine malignancy known for its aggressive nature and high mortality rate, surpassing that of malignant melanoma [1]. Usually, MCC presents with a non-tender, firm, red-colored nodule in the head and neck region of elderly, white males [2]. Other risk factors include ultraviolet exposure, immunosuppression, and history of malignancy [1]. Most MCC cases occur when Merkel cell polyomavirus (MCPyV) is integrated into the host genome [1]. MCPyV-negative MCC cases are associated with ultraviolet radiation genetic damage [3]. Although MCC begins as an indolent growth, its course quickly turns aggressive due to lymph node (LN) invasion and distant metastasis [4]. Given its rapid nature, MCC should be considered as a differential diagnosis of a patient presenting with a nonspecific nodule without typical risk factors.

## Case report

**Figure 1:**
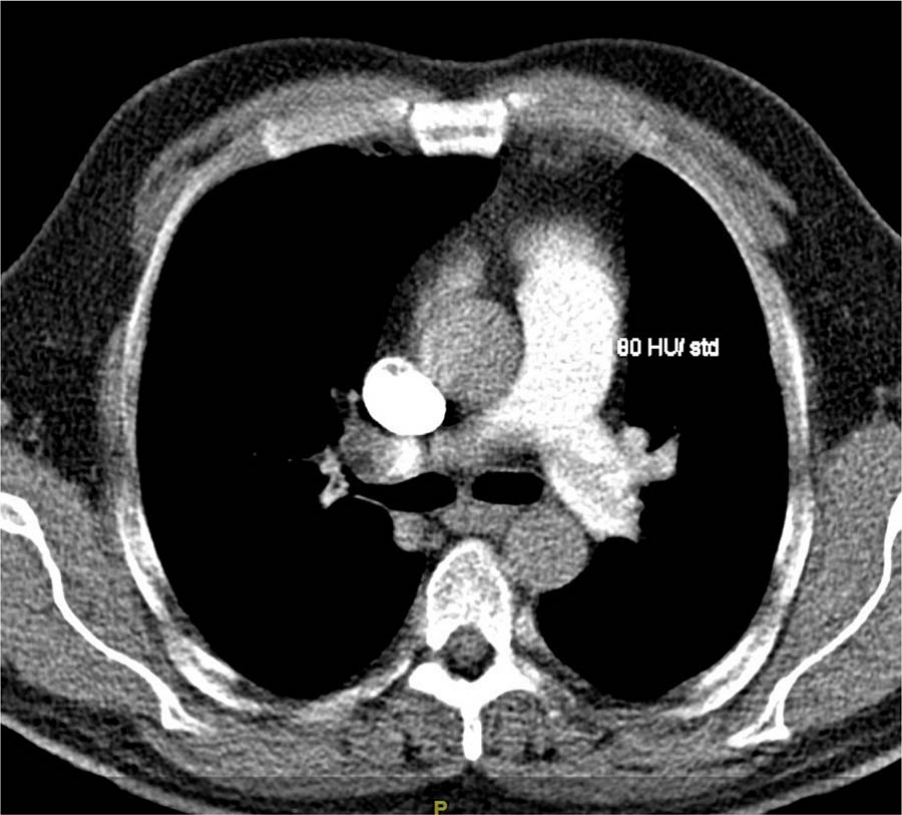
Main pulmonary artery saddle embolus with extension into lobar, segmental, and subsegmental branches of both lungs.

A 48-year-old Caucasian male with a past medical history of Type II Diabetes Mellitus, hyperlipidemia, hypertension, inguinal and umbilical hernias, and left groin mass presented to the Emergency Department (ED) with chest pain, dyspnea, vague epigastric pain, and left extremity swelling. He is a nonsmoker and works as a car retailer. Computed Tomography (CT) angiography showed bilateral saddle pulmonary embolism (PE) with right heart strain (Fig. 1). Patient was admitted to the Intensive Care Unit for management and was started on a heparin drip protocol. A Deep Vein Thrombosis (DVT) scan of his left lower extremity showed occlusive thrombus of the common femoral, femoral saphenous junction, superficial femoral, and popliteal veins. Chest x-ray revealed patchy opacities in the left midlung and mass-like consolidation at the left hilum.

**Figure 2:**
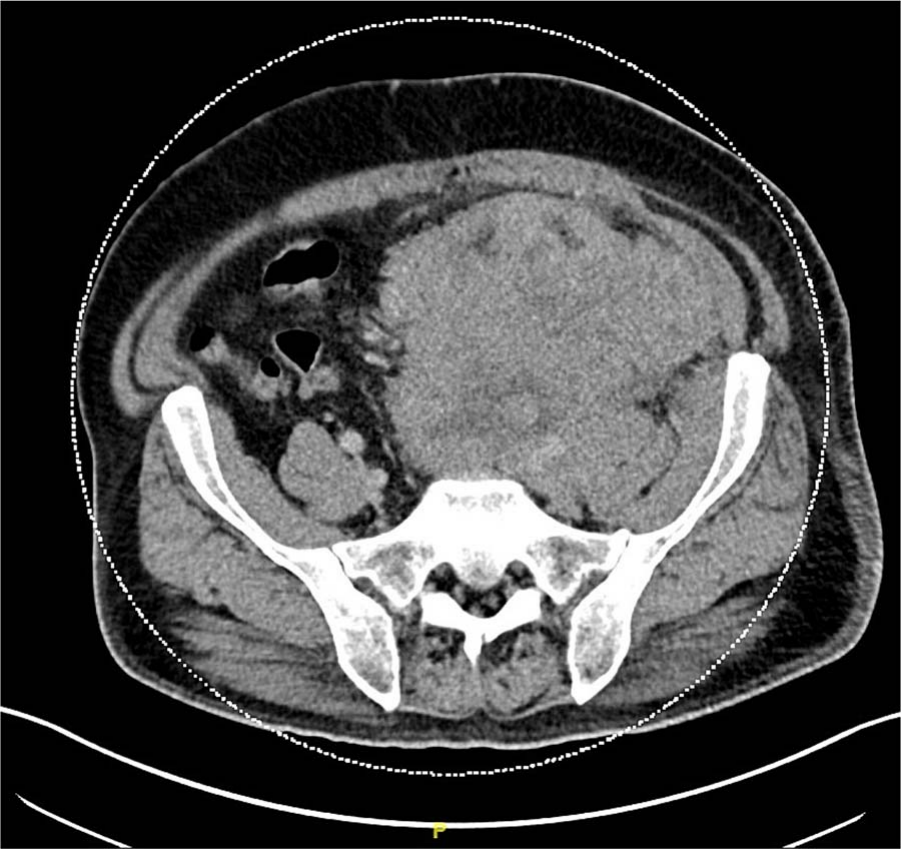
Left hemipelvic heterogeneous mass encasing the left common iliac and left external iliac vasculature.

The patient reported that he had groin swelling for three years. CT showed a retroperitoneal heterogeneous 14 × 13 × 18 cm mass encasing the left common and external iliac vasculatures (Fig. 2). A large left inguinal heterogeneous mass was also detected. Left groin mass biopsy showed intermediate size tumor cells with very high N:C ratio. Chromatin was delicate with a salt and pepper appearance, nucleoli were small or absent, and mitoses were identified throughout the tumor. Tumor infiltrating lymphocytes were nonbrisk, and there was extensive hyaline fibrosis and focal necrosis. Flow cytometry study expressed CD56. Immunohistochemical stains revealed tumor cells positive for synaptophysin, chromogranin with focal positivity for AE1AE3, CK7, and CK20 (focal paranuclear dot-like), supporting a diagnosis of MCC. Inguinal LN biopsy confirmed cTxN1M1a MCC with an occult primary lesion.

Post-discharge, Positron Emission Tomography/CT revealed two predominant tumor masses, including a left 16 × 16 × 18 cm pelvic/retroperitoneal mass and a left 8.3 × 5.1 × 9.0 cm inguinal LN conglomerate.

**Figure 3:**
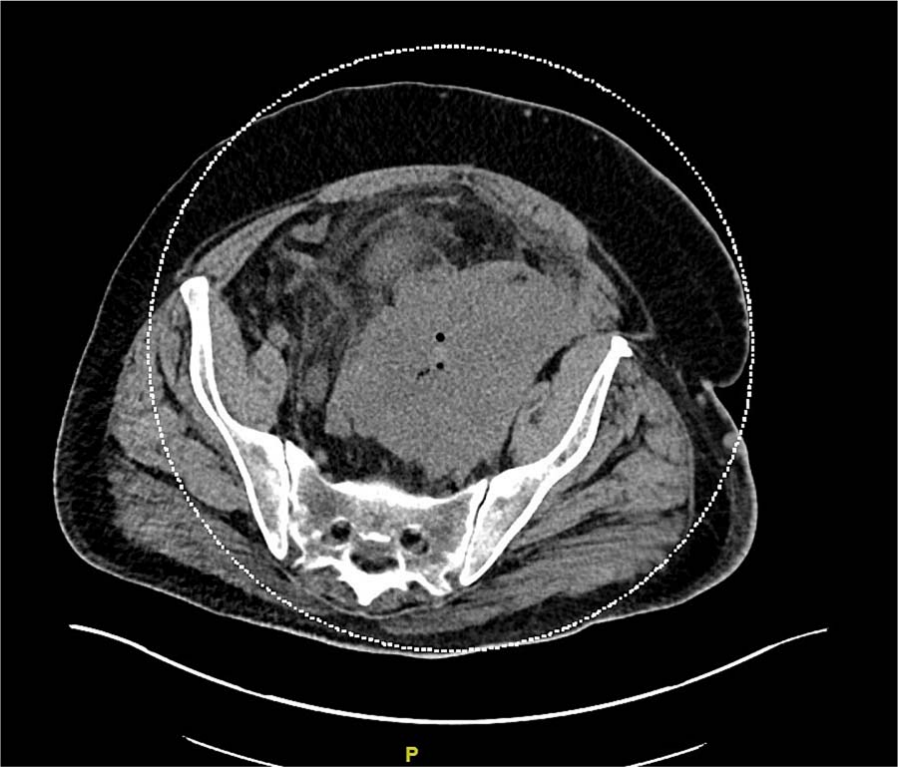
Left pelvic mass obstructing left ureter and invading the left pelvic sidewall, descending colon, sigmoid colon, and urinary bladder.

The patient underwent four cycles of nivolumab. CT showed a decrease in the size of the pelvic mass to 13 × 9 cm (Fig. 3). However, the left inguinal node conglomerate remained unchanged. The patient was then offered intensity-modulated radiation therapy (IMRT) to a dose of 6000–6600 cGY in 30–33 fractions for the left inguinal LN mass. Since IMRT could not be used for the left pelvic mass due to proximity to the colon, nivolumab was used.

## Discussion

Metastatic MCC with an unknown primary (MCC-UP) has a better prognosis than known primary (MCC-KP). It has been reported that patients with nodal MCC without a discernible primary lesion are about twice as likely to survive compared with MCC-KP [5]. A recent clinical trial demonstrated that nivolumab, a monoclonal antibody that targets PD-1, is a feasible adjuvant immunotherapy for MCC [6].

Our patient presented to the ED with risk factors and symptoms strongly suggesting DVT and PE. His overdue inguinal biopsy was performed due to concern for a hypercoagulable state secondary to malignancy.

The workup of our patient showed an occult primary lesion and metastasis to LN in the left inguinal chain and pelvis, classifying it as MCC-UP. In MCC-UP, it is hypothesized that regression of the primary lesion is immune-mediated, involving enhanced tumor immunogenicity [6].

Our patient had only two risk factors, race and sex, for the development of MCC. Although MCC is more common in older people, some cases have shown to present in younger individuals like our patient [7]. In fact, about 5% of MCC cases occur in patients younger than 50 years old [7]. It is important to include MCC as a differential in patients who do not have many risk factors.
